# Can glypican-3 be a disease-specific biomarker?

**DOI:** 10.1186/s40169-017-0146-5

**Published:** 2017-05-16

**Authors:** Chaolei Chen, Xiaomin Huang, Zhaojian Ying, Dengmin Wu, Yani Yu, Xiangdong Wang, Chengshui Chen

**Affiliations:** Department of Pulmonary Medicine, The First Affiliated Hospital of Wenzhou Medical University, Wenzhou, China

**Keywords:** Glypican-3, Cancer, Biomarkers, Disease, Therapy

## Abstract

**Background:**

Glypican-3 (GPC3) is a cell surface-bound proteoglycan which has been identified as a potential biomarker candidate in hepatocellular carcinoma, lung carcinoma, severe pneumonia, and acute respiratory distress syndrome (ARDS). The aim of our review is to evaluate whether GPC3 has utility as a disease-specific biomarker, to discuss the potential involvement of GPC3 in cell biology, and to consider the changes of GPC3 gene and protein expression and regulation in hepatocellular carcinoma, lung cancer, severe pneumonia, and ARDS.

**Results:**

Immunohistochemical studies have suggested that over-expression of GPC3 is associated with a poorer prognosis for hepatocellular carcinoma patients. Expression of GPC3 leads to an increased apoptosis response in human lung carcinoma tumor cells, and is considered to be a candidate lung tumor suppressor gene. Increased serum levels of GPC3 have been demonstrated in ARDS patients with severe pneumonia.

**Conclusions:**

Glypican-3 could be considered as a clinically useful biomarker in hepatocellular carcinoma, lung carcinoma, and ARDS, but further research is needed to confirm and expand on these findings.

## Introduction

Acute respiratory distress syndrome (ARDS) is a life-threatening syndrome characterized by the acute onset of pulmonary edema of non-cardiogenic origin, along with bilateral pulmonary infiltrates and reduction in respiratory system compliance in the seriously ill patient. ARDS is a complex response of the lung to direct and indirect insults associated with high morbidity and mortality, with current treatments mainly being supportive, without accurate targeted therapies [1, 2]. Directions of research are concentrating on identifying potential biomarkers or genetic markers to facilitate diagnosis, and to assist in prediction of outcome and treatment response. Recently, our group found that the serum level of Glypican-3 (GPC3), a proteoglycan anchored to cell surface by glycosyl-phosphatidylinositol, was significantly higher in the circulation of patients with severe pneumonia, as compared with healthy control, and increased even more significantly in patients with severe pneumonia accompanied with ARDS than those with severe pneumonia alone [3]. We proposed that the circulating level of glypican-3 may correlate with the severity of pneumonia as potential biomarker to predict the occurrence of ARDS. GPC3 has been recently reported and suggested as a novel potential oncofetal biomarker for diagnosis in a number of cancer diseases such as hepatocellular carcinoma [4]. It has previously been demonstrated that GPC3 was over-expressed in human hepatocellular carcinoma measured by cDNA microarrays, and GPC3 protein was found in serum from 40% of patients with hepatocellular carcinoma, but not in serum from patients with liver cirrhosis, chronic hepatitis, and healthy donors [4]. Therefore, GPC3 was proposed as a useful tumor marker for cancer-diagnosis for patients with hepatocellular carcinoma. Specific role of GPC3 in cancer and inflammatory disease at different times seems to have a clear and reasonable disease control, e.g., severe pneumonia with or without ARDS, or virus-infected patients with hepatocellular carcinoma compared with other liver diseases [3, 4]. The significant increases in expression of GPC3 in hepatocellular carcinoma and ARDS raise the question whether GPC3 has utility as a biomarker of disease or disease severity [5–8]. The present commentary calls for further research into the molecular biology, disease-specific associations, and potential value as a biomarker of GPC3.

## Gene organization of GPC3 and its family

Glypican-3, also called OCI-5, DGSX, GTR2-2, MXR7, SDYS, SGB, SGBS, and SGBS1, was identified in a rare undifferentiated epithelial cell line OCI-5 [9]. It is a cell surface heparan sulfate proteoglycan belonging to the glypican-related integral membrane proteoglycan family [10], which includes six members (GPC1–GPC6). According to the homogeneity and heterogeneity of gene sequences, these six members are divided into two subfamilies; one group is GPC3 and GPC5 which show 43% sequence homology, while the other group contains GPC1, GPC2, GPC4, and GPC6 [11]. *GPC3* is encoded at chromosome Xp26 adjacent to GPC4, and spans more than 500 kilobases. Four isoforms have been reported, of which isoform 2 (GenBank Accession No.: NP_004475), which encodes a 70-kDa precursor core protein with 580 amino acids, is the most commonly expressed [12].

Glypicans are composed of a 60–70 kDa size membrane-associated core protein, a variable number of heparan sulfate glycosaminoglycan polysaccharide side chains, and a glycosyl-phosphatidylinositol linkage. The core protein is predicted to form a conserved globular tertiary structure because of several intramolecular disulfide bridges caused by 14 conserved cysteine residues domains. Heparan sulfate chains bind to serine residues of core protein carboxyl terminal protein [13], and approximate the core protein to the cell membrane. A glycosyl-phosphatidylinositol linkage anchor becomes the final connecting link between the core protein and the cell membrane. GPC3 has one 40-kDa amino terminal protein and one 30-kDa membrane-bound carboxyl terminal protein. The amino acid sequences for the two different terminal proteins are Arg358 and Ser359, which can be enzymatically lyzed releasing a soluble form of GPC3 (sGPC3). sGPC3 can be specifically detected in the circulating blood of patients with hepatocellular carcinoma [14]. Secreted GPC3 can also be cleaved by notum, a secreted enzyme, into a released form in the extracellular environment [15]. The notum’s cleavage site and glycosyl-phosphatidylinositol linkage-phospholipase C, the glycosyl-phosphatidylinositol linkage anchor of GPC3, are two distinct soluble GPC3 forms, each having different functions.

## Roles of GPC3 in diseases

**Fig. 1:**
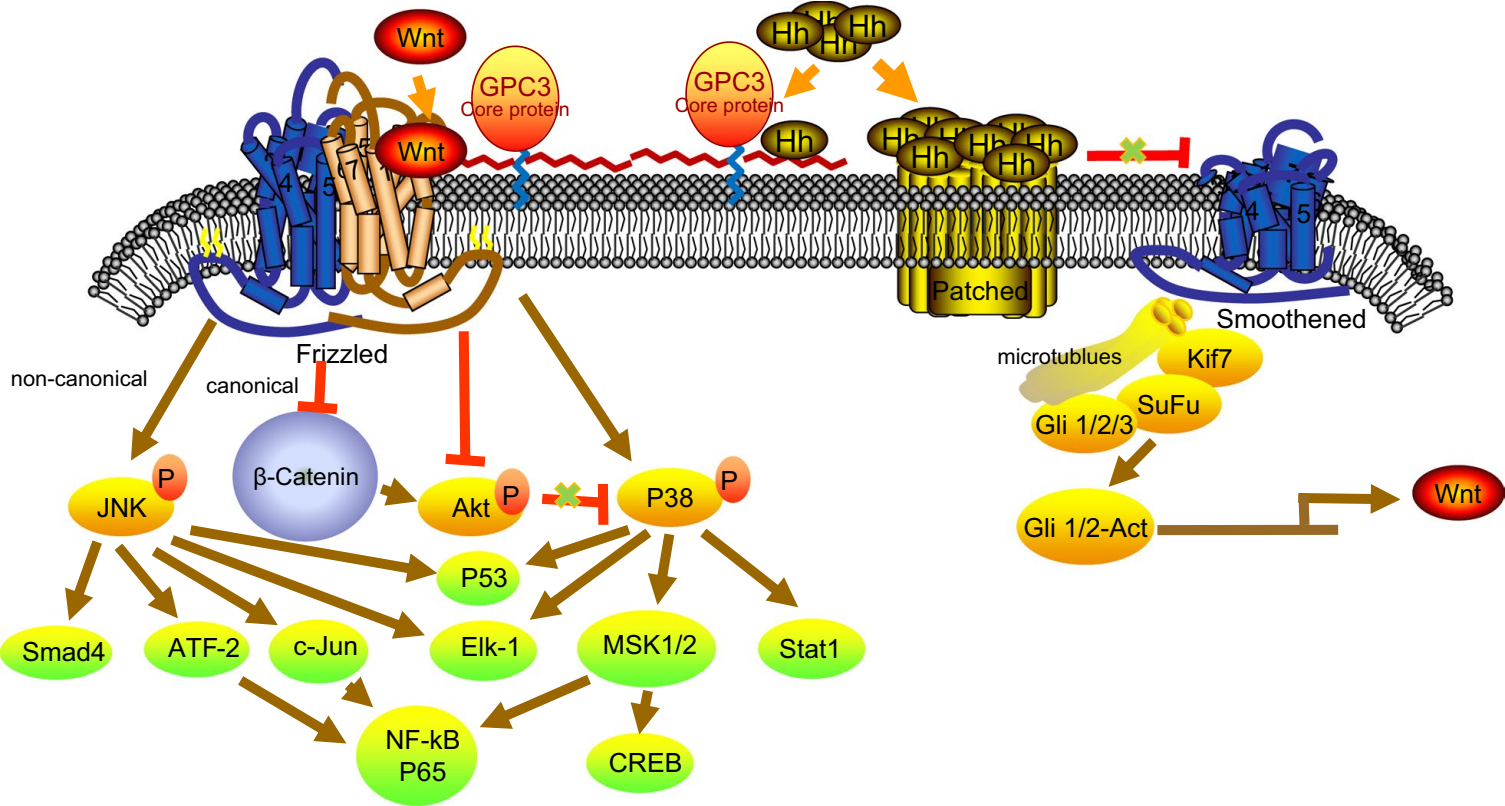
Positive and negative effects of GPC3 on its two main signaling pathways. In the Wnt signaling pathway (*left*), GPC3 suppresses canonical Wnt signaling pathway (Wnt/β-catenin) while motivating the activation of non-canonical Wnt signaling pathway (Wnt/JNK). In the Hedgehog (Hh) signaling pathway (*right*), this signaling pathway can be triggered by combination of Hh and the receptor Patched (Ptc) which leads to the inhibition of Ptc on Smoothened. GPC3 competes with Ptc for Hh binding

Glypican-3 gene is weakly expressed in the placenta and lung, kidney, ovarian, breast, and skin tissue, but not in normal adult liver, heart, brain, spleen, stomach, intestine, testicles, and bladder tissue [16]. GPC3 plays important roles in tissue morphogenesis and homeostasis during development, e.g., developmental outgrowth and dysplastic kidneys of Simpson-Golabi-Behmel syndrome [17], via Wnt/JNK signaling [18] or FGF signaling pathways [19], in the processes of chondrogenesis and osteogenesis via the morphogenetic protein signaling pathway [20], and several malignancies as a cancer suppressor gene through a suppression of PI3K/Akt pathways and a stimulation of P38/MAPK pathway [21] (Fig. 1). GPC3 contributes to cell proliferation and survival, and regulates breast cancer cell growth [22].

Increasing evidence suggests soluble GPC3 as a novel diagnostic candidate marker in hepatocellular carcinoma [13]. GPC3 is expressed in 95% of hepatocellular carcinomas, but not in neuroendocrine tumor metastatic to the liver, and cholangiocarcinoma, measured by liver cancer tissue microarrays which were constructed from hepatocellular carcinoma, neuroendocrine tumor, and cholangiocarcinoma samples [23]. The high positive rate of GPC3 in hepatocellular carcinoma indicates the specificity of GPC3 in hepatocyte-origin carcinogenesis. GPC3 was considered as important as alpha-fetoprotein in a fast and effective cell sorting strategy to specifically identify hepatocellular carcinoma circulating cells [24]. The positive rate of hepatocellular carcinoma circulating cells was above 90% in combination of GPC3 with alpha-fetoprotein. The hepatocellular carcinoma cells positive to both GPC3 and cytokeratin 19 were found to have the highest risk of multifocality, microvascular invasion, regional lymph node involvement, shortest recurrence time, and distant metastasis in a retrospective study of immunohistochemical staining [25]. The combination of GPC3 and cytokeratin 19 expression in the cancer tissue was suggested as an independent prognostic indicator in patients with hepatocellular carcinoma.

Glypican-3 was recently suggested to be a critical part of molecular mechanisms by which the proliferation and invasion of hepatocellular carcinoma are regulated and controlled. MicroRNA-133b is a small non-coding RNA which targets Sirt1s, suppressing its expression in hepatocellular carcinoma cells, increasing the proliferation and invasion of hepatocellular carcinoma cells through the activation of E-cadherin expression, and repressing expression of GPC3 and the anti-apoptotic proteins (Bcl-2, Bcl-xL, and Mcl-1) [26]. It is questioned whether the GPC3/Wnt β-catenin signal pathway is miR-133b/Sirt1-specific regulation or is the hepatocellular carcinoma cell-specific and/or dominated mechanism. A clinical trial of codrituzumab, a humanized monoclonal antibody against GPC3, was recently performed in a randomized phase II trial in advanced hepatocellular carcinoma patients who had failed prior systemic therapy [27]. It was proposed that GPC3 could interact with CD16/FcγRIIIa and trigger antibody-dependent cytotoxicity in hepatocellular carcinoma cells. Patients with vascular invasion and/or extra-hepatic metastasis were treated with sorafenib and then with codrituzumab or placebo. This particular study with 125 patients with codrituzumab failed to show any significant improvement of the median progression-free survival and overall survival, even though those patients had high CD16/FcγRIIIa expression on peripheral immune cells and GPC3 expression in the tumor.

Oligonucleotide microarray analysis demonstrated that GPC3 is over-expressed in tissues harvested from smokers with lung adenocarcinoma [28]. The importance of GPC3 was furthermore validated in lung cancer cell lines. It was proposed that the over-expression of GPC3 may be induced by smoking, although no direct evidence to support this has been reported. High expression of GPC3 was noticed in lung cancer tissues, particularly in lung squamous cell carcinoma. GPC3 protein and mRNA expression were positive in 55% of squamous cell carcinoma versus 8% of adenocarcinoma, but negative in normal lung tissues [28]. The suggestion that GPC3 was suggested as a potential candidate marker for early detection of lung squamous cell carcinoma should be questioned, since about half of patients with lung squamous cell carcinoma have negative GPC3 staining, and because it is hard to access the lung tissue at the early stage of the disease.

When comparing the sensitivity of tissue markers using immunostaining [29], the positive staining of p63 was about 99% in both lung squamous cell carcinoma and adenocarcinoma, followed by high-molecular-weight cytokeratin (HMCK) (97%), cytokeratin 5/6 (93%), SRY-related HMG-box 2 (80%), thrombomodulin (79%), desmocollin-3 (72.7%), S100A7 (71%), S100A2 (63.3%), and GPC3 (47%), while desmocollin-3 in lung squamous cell carcinoma alone was 100%, followed by cytokeratin 5/6 (98%), SRY-related HMG-box 2 (96%), GPC3 (92%), S100A7 (87%), thrombomodulin (80%), S100A2 (65%), p63 (52%), and HMCK (33%). Thyroid transcription factor-1 (TTF-1) expression was observed in 87.4% of lung adenocarcinoma cases and 2.0% of squamous cell lung carcinoma cases. When analyzing only poorly differentiated tumors, HMCK was the most sensitive marker for squamous cell lung carcinoma (100%), followed by p63 (97.8%), CK5/6 (87.0%), Sox2 (71.7%), thrombomodulin (58.7%), desmocollin-3 (52.2%), S100A2 (50%), glypican-3 (45.7%), and S100A7 (45.7%). Desmocollin-3 was the most specific marker for poorly differentiated squamous cell lung carcinoma (100%), followed by CK5/6 (98.3%), glypican-3 (94.8%), Sox2 (94.8%), S100A2 (81%), S100A7 (75.9%), thrombomodulin (72.4%), p63 (48.3%), and HMCK (36.8%). The study by Tsuta et al. demonstrated that a number of molecules could be altered according to disease subtypes, differentiations, durations, and severities. The cytokeratin 5/6 was suggested as the best marker for differentiating lung squamous carcinoma and lung adenocarcinoma, although GPC3 and others were also differentially expressed compared with controls. In addition, an early study demonstrated an increased apoptosis response caused by ectopic expression of GPC3 in human lung carcinoma tumor cell, and GPC3 would be a candidate lung tumor suppressor gene [30], although little has been known on accurate mechanisms of GPC3 in the carcinogenesis of lung cancer.

## Concerns on disease specificity

The specificity of GPC3 as disease biomarkers should be carefully examined and validated in large cohorts, in a comparison among multiple diseases, and in cases of different stages, duration, and severity. GPC3 was firstly reported as a disease-specific biomarker for ARDS [3]. GPC3 was identified by integrating proteomic profiles of inflammatory mediators with clinical bioinformatics [7]. In the research by Chen et al., plasma was collected from the healthy persons as controls or from patients with severe pneumonia infected by bacteria or from patients with severe pneumonia-associated ARDS on day of the admission, day 3, and day 7 [3]. Expression of GPC3 in peripheral circulation of severe pneumonia-associated ARDS patients progressively increased over time (admission day, day 3, day 7) compared with healthy persons or severe pneumonia patients alone. The investigators further showed that circulating levels of GPC3 were increased in ARDS induced by severe pneumonia as a model of infection-dominated disease, as well as in ARDS induced by acute pancreatitis as a model of non-infection-based disease. It seems that altered GPC3 may be more dependent upon the disease stage and severity, rather than the properties of associated pathogens or initiates. In acute liver injury models induced by lipopolysaccharide, expression of GPC3 genes increased over time after induction, and even more significantly when bone marrow-derived mesenchymal stem cells were transplanted [31].

**Fig. 2:**
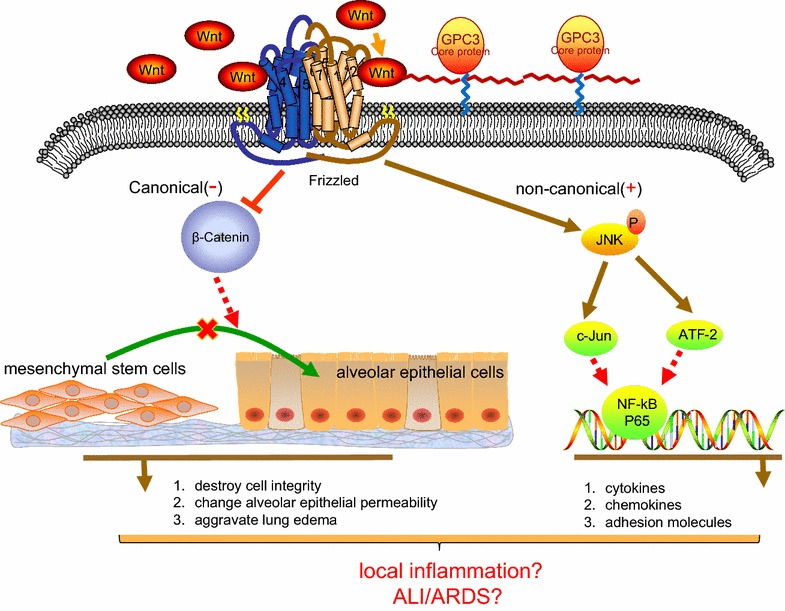
Potential mechanisms of GPC3 in acute lung injury. Wnt/β-catenin signaling pathway shows its ability to transform the mesenchymal stem cells to alveolar epithelial cells. The suppression of Wnt/β-catenin signaling pathway by GPC3 may destroy cell integrity, change alveolar epithelial permeability, and aggravate lung edema. On the other hand, GPC3 can enhance Wnt/JNK signaling pathway which may create an increase transcription of NF-κB

The mechanism by which GPC3 influences ARDS is unknown. Based on the known GPC3-related signaling pathways [32–34], we propose a possible hypothesis that the GPC3-Wnt pathway may play a significant role in the development and progression of ARDS (Fig. 2). GPC3 has opposite effects on the canonical and non-canonical Wnt signaling pathways, suppressing canonical Wnt/β-catenin signaling while activating non-canonical Wnt signaling pathway (Wnt/JNK) [18]. The Wnt/β-catenin signaling pathway can induce transformation of mesenchymal stem cells to alveolar epithelial cells [35]. Thus, a suppression of Wnt/β-catenin signaling pathway by GPC3 may destroy cell integrity, change alveolar epithelial permeability, and aggravate lung edema. On the other hand, an enhancement of Wnt/JNK signaling pathway caused by GPC3 may induce NF-κB activation, which is a classic proinflammatory transcription factor in many inflammatory diseases including ARDS [36]. We therefore hypothesize that GPC3 may be involved in the tissue/cell-associated auto-defensive processes, repair and recovery, or regeneration.

## Conclusion

Glypican-3 is involved in the inhibition of cell proliferation and modulation of cell survival during development process of the organ. It may have value as a biomarker of hepatocellular carcinoma, lung carcinoma, severe pneumonia, and ARDS. GPC3 was recently selected and proposed as a biomarker in patients with severe pneumonia and ARDS [3]. The potential of GPC3 as a disease biomarker needs further study in different diseases in particular in lung diseases, such as ARDS, and in cancers including lung and liver cancer. These studies should involve large cohorts with a variety of disease durations and severity, and in the case of ARDS, of inducing factors. The biological functions and roles of GPC3 require further investigation to understand the molecular mechanisms underpinning the associations of GPC3 with disease. We aim to further evaluate the utility of GPC3 as a biomarker, in order to enable producing more precise and accurate medicine.

## References

[1] Definition Task Force ARDS, Ranieri VM, Rubenfeld GD, Thompson BT, Ferguson ND, Caldwell E, Fan E, Camporota L, Slutsky AS (2012). Acute respiratory distress syndrome: the Berlin definition. JAMA.

[2] Bellani G, Laffey JG, Pham T, Fan E, Brochard L, Esteban A, Gattinoni L, van Haren F, Larsson A, McAuley DF, Ranieri M, Rubenfeld G, Thompson BT, Wrigge H, Slutsky AS, Pesenti A, Investigators LS, Group ET (2016). Epidemiology, patterns of care, and mortality for patients with acute respiratory distress syndrome in intensive care units in 50 countries. JAMA.

[3] Chen C, Shi L, Li Y, Wang X, Yang S (2016). Disease-specific dynamic biomarkers selected by integrating inflammatory mediators with clinical informatics in ARDS patients with severe pneumonia. Cell Biol Toxicol.

[4] Nakatsura T, Yoshitake Y, Senju S, Monji M, Komori H, Motomura Y, Hosaka S, Beppu T, Ishiko T, Kamohara H, Ashihara H, Katagiri T, Furukawa Y, Fujiyama S, Ogawa M, Nakamura Y, Nishimura Y (2003). Glypican-3, overexpressed specifically in human hepatocellular carcinoma, is a novel tumor marker. Biochem Biophys Res Commun.

[5] Wu X, Chen L, Wang X (2014). Network biomarkers, interaction networks and dynamical network biomarkers in respiratory diseases. Clin Transl Med.

[6] Truedsson M, Malm J, Barbara Sahlin K, Bugge M, Wieslander E, Dahlback M, Appelqvist R, Fehniger TE, Marko-Varga G (2016). Biomarkers of early chronic obstructive pulmonary disease (COPD) in smokers and former smokers. Protocol of a longitudinal study. Clin Transl Med.

[7] Wu D, Wang X (2015). Application of clinical bioinformatics in lung cancer-specific biomarkers. Cancer Metastasis Rev.

[8] Wang X, Ward PA (2012). Opportunities and challenges of disease biomarkers: a new section in the journal of translational medicine. J Transl Med.

[9] Filmus J, Church JG, Buick RN (1988). Isolation of a cDNA corresponding to a developmentally regulated transcript in rat intestine. Mol Cell Biol.

[10] Pilia G, Hughes-Benzie RM, MacKenzie A, Baybayan P, Chen EY, Huber R, Neri G, Cao A, Forabosco A, Schlessinger D (1996). Mutations in GPC3, a glypican gene, cause the Simpson-Golabi-Behmel overgrowth syndrome. Nat Genet.

[11] Bulow HE, Hobert O (2006). The molecular diversity of glycosaminoglycans shapes animal development. Annu Rev Cell Dev Biol.

[12] Ho M, Kim H (2011). Glypican-3: a new target for cancer immunotherapy. Eur J Cancer.

[13] Hippo Y, Watanabe K, Watanabe A, Midorikawa Y, Yamamoto S, Ihara S, Tokita S, Iwanari H, Ito Y, Nakano K, Nezu J, Tsunoda H, Yoshino T, Ohizumi I, Tsuchiya M, Ohnishi S, Makuuchi M, Hamakubo T, Kodama T, Aburatani H (2004). Identification of soluble NH2-terminal fragment of glypican-3 as a serological marker for early-stage hepatocellular carcinoma. Cancer Res.

[14] De Cat B, Muyldermans SY, Coomans C, Degeest G, Vanderschueren B, Creemers J, Biemar F, Peers B, David G (2003). Processing by proprotein convertases is required for glypican-3 modulation of cell survival, Wnt signaling, and gastrulation movements. J Cell Biol.

[15] Traister A, Shi W, Filmus J (2008). Mammalian Notum induces the release of glypicans and other GPI-anchored proteins from the cell surface. Biochem J.

[16] Iglesias BV, Centeno G, Pascuccelli H, Ward F, Peters MG, Filmus J, Puricelli L, de Kier Joffe EB (2008). Expression pattern of glypican-3 (GPC3) during human embryonic and fetal development. Histol Histopathol.

[17] Pellegrini M, Pilia G, Pantano S, Lucchini F, Uda M, Fumi M, Cao A, Schlessinger D, Forabosco A (1998). Gpc3 expression correlates with the phenotype of the Simpson-Golabi-Behmel syndrome. Dev Dyn.

[18] Song HH, Shi W, Xiang YY, Filmus J (2005). The loss of glypican-3 induces alterations in Wnt signaling. J Biol Chem.

[19] Grisaru S, Cano-Gauci D, Tee J, Filmus J, Rosenblum ND (2001). Glypican-3 modulates BMP- and FGF-mediated effects during renal branching morphogenesis. Dev Biol.

[20] Dwivedi PP, Lam N, Powell BC (2013). Boning up on glypicans—opportunities for new insights into bone biology. Cell Biochem Funct.

[21] Pan Z, Chen C, Long H, Lei C, Tang G, Li L, Feng J, Chen F (2013). Overexpression of GPC3 inhibits hepatocellular carcinoma cell proliferation and invasion through induction of apoptosis. Mol Med Rep.

[22] Buchanan C, Stigliano I, Garay-Malpartida HM, Rodrigues Gomes L, Puricelli L, Sogayar MC, de Kier Bal, Joffe E, Peters MG (2010). Glypican-3 reexpression regulates apoptosis in murine adenocarcinoma mammary cells modulating PI3K/Akt and p38MAPK signaling pathways. Breast Cancer Res Treat.

[23] Jin M, Zhou X, Yearsley M, Frankel WL (2016). Liver Metastases of Neuroendocrine Tumors Rarely Show Overlapping Immunoprofile with Hepatocellular Carcinomas. Endocr Pathol.

[24] Chen L, Wu LL, Zhang ZL, Hu J, Tang M, Qi CB, Li N, Pang DW (2016). Biofunctionalized magnetic nanospheres-based cell sorting strategy for efficient isolation, detection and subtype analyses of heterogeneous circulating hepatocellular carcinoma cells. Biosens Bioelectron.

[25] Feng J, Zhu R, Chang C, Yu L, Cao F, Zhu G, Chen F, Xia H, Lv F, Zhang S, Sun L (2016). CK19 and glypican 3 expression profiling in the prognostic indication for patients with HCC after surgical resection. PLoS ONE.

[26] Tian Z, Jiang H, Liu Y, Huang Y, Xiong X, Wu H, Dai X (2016). MicroRNA-133b inhibits hepatocellular carcinoma cell progression by targeting Sirt1. Exp Cell Res.

[27] Abou-Alfa GK, Puig O, Daniele B, Kudo M, Merle P, Park JW, Ross P, Peron JM, Ebert O, Chan S, Poon TP, Colombo M, Okusaka T, Ryoo BY, Minguez B, Tanaka T, Ohtomo T, Ukrainskyj S, Boisserie F, Rutman O, Chen YC, Xu C, Shochat E, Jukofsky L, Reis B, Chen G, Di Laurenzio L, Lee R, Yen CJ (2016). Randomized phase II placebo controlled study of codrituzumab in previously treated patients with advanced hepatocellular carcinoma. J Hepatol.

[28] Aviel-Ronen S, Lau SK, Pintilie M, Lau D, Liu N, Tsao MS, Jothy S (2008). Glypican-3 is overexpressed in lung squamous cell carcinoma, but not in adenocarcinoma. Mod Pathol.

[29] Tsuta K, Tanabe Y, Yoshida A, Takahashi F, Maeshima AM, Asamura H, Tsuda H (2011). Utility of 10 immunohistochemical markers including novel markers (desmocollin-3, glypican 3, S100A2, S100A7, and Sox-2) for differential diagnosis of squamous cell carcinoma from adenocarcinoma of the Lung. J Thorac Oncol.

[30] Kim H, Xu GL, Borczuk AC, Busch S, Filmus J, Capurro M, Brody JS, Lange J, D’Armiento JM, Rothman PB, Powell CA (2003). The heparan sulfate proteoglycan GPC3 is a potential lung tumor suppressor. Am J Respir Cell Mol Biol.

[31] Cai Y, Zou Z, Liu L, Chen S, Chen Y, Lin Z, Shi K, Xu L, Chen Y (2015). Bone marrow-derived mesenchymal stem cells inhibits hepatocyte apoptosis after acute liver injury. Int J Clin Exp Pathol.

[32] Capurro MI, Xiang YY, Lobe C, Filmus J (2005). Glypican-3 promotes the growth of hepatocellular carcinoma by stimulating canonical Wnt signaling. Cancer Res.

[33] Stigliano I, Puricelli L, Filmus J, Sogayar MC, de Kier Bal, Joffe E, Peters MG (2009). Glypican-3 regulates migration, adhesion and actin cytoskeleton organization in mammary tumor cells through Wnt signaling modulation. Breast Cancer Res Treat.

[34] Capurro M, Martin T, Shi W, Filmus J (2014). Glypican-3 binds to Frizzled and plays a direct role in the stimulation of canonical Wnt signaling. J Cell Sci.

[35] Cai SX, Liu AR, Chen S, He HL, Chen QH, Xu JY, Pan C, Yang Y, Guo FM, Huang YZ, Liu L, Qiu HB (2015). Activation of Wnt/beta-catenin signalling promotes mesenchymal stem cells to repair injured alveolar epithelium induced by lipopolysaccharide in mice. Stem Cell Res Ther.

[36] Lee CY, Yang JJ, Lee SS, Chen CJ, Huang YC, Huang KH, Kuan YH (2014). Protective effect of Ginkgo biloba leaves extract, EGb761, on endotoxin-induced acute lung injury via a JNK- and Akt-dependent NFkappaB pathway. J Agric Food Chem.

